# Molecular detection of *Haemoproteus columbae* Kruse, 1890 (Haemospororida: Haemoproteidae) in *Columba livia* Gmelin, 1789 (Columbiformes: Columbidae) in southern Brazil

**DOI:** 10.1590/S1984-29612025027

**Published:** 2025-06-13

**Authors:** Carolina Caetano dos Santos, Natália Soares Martins, Nilséia Feijó da Silva, Julia Somavilla Lignon, Kauê Rodriguez Martins, Oluwagbemiga Ademola Dada, Rodrigo Casquero Cunha, Fábio Raphael Pascoti Bruhn, Diego Moscarelli Pinto, Felipe Geraldo Pappen, Carolina Silveira Mascarenhas, Nara Amélia da Rosa Farias

**Affiliations:** 1 Laboratório de Parasitologia XIII, Departamento de Microbiologia e Parasitologia, Instituto de Biologia, Universidade Federal de Pelotas – UFPel, Pelotas, RS, Brasil; 2 Laboratório do Grupo de Estudos em Enfermidades Parasitárias, Departamento de Veterinária Preventiva, Universidade Federal de Pelotas – UFPel, Pelotas, RS, Brasil; 3 Laboratório de Epidemiologia Veterinária, Departamento de Veterinária Preventiva, Universidade Federal de Pelotas – UFPel, Pelotas, RS, Brasil; 4 Laboratório de Biologia Molecular Veterinária, Departamento de Veterinária Preventiva, Universidade Federal de Pelotas – UFPel, Pelotas, RS, Brasil; 5 Department of Agricultural Science, Adeyemi Federal University of Education, Ondo, Nigeria; 6 Pesquisador independente, Capão do Leão, RS, Brasil

**Keywords:** Blood smear, Haemosporidian, PCR, pigeons, Rio Grande do Sul, Esfregaço sanguíneo, Haemosporídio, PCR, pombos, Rio Grande do Sul

## Abstract

The aim of this study is to describe the molecular detection of *Haemoproteus columbae* Kruse, 1890 (Haemospororida: Haemoproteidae) in *Columba livia* Gmelin, 1789 (Columbiformes: Columbidae) in southern Brazil, and to determine the prevalence of the haemosporidian by analyzing the infection in relation to birds’ age, sex, and place of origin. Blood samples were collected from 57 birds captured in the municipalities of ​​Pelotas and Rio Grande, in the state of Rio Grande do Sul, Brazil, between May and November 2022. Microscopic examination and Polymerase Chain Reaction (PCR) based on the mitochondrial cytochrome b gene (*CytB*) revealed that the prevalence of the haemosporidian was 92.98% (53/57). In the sequencing analysis, the samples showed 98.87-99.08% similarity to the species *H. columbae* and a new lineage was recorded. No differences in infection rates were observed in relation to age, sex, or place of origin of the birds.

## Resumo

O objetivo deste estudo é descrever a detecção molecular de *Haemoproteus columbae* Kruse, 1890 (Haemospororida: Haemoproteidae) em *Columba livia* Gmelin, 1789 (Columbiformes: Columbidae) no sul do Brasil, e determinar a frequência do haemosporídio, analisando-se a infecção em relação à idade, sexo e local de origem das aves. De maio a novembro de 2022, foram coletadas amostras de sangue de 57 aves capturadas nos municípios de Pelotas e Rio Grande, no estado do Rio Grande do Sul, Brasil. O exame microscópico e a Reação em Cadeia da Polimerase (PCR), baseada no gene do citocromo b mitocondrial (*CytB*), revelaram que a taxa de frequência do haemosporídio foi de 92,98% (53/57). Na análise de sequenciamento, as amostras apresentaram 98,87-99,08% de similaridade com a espécie *H. columbae* e uma nova linhagem foi registrada. Não foram observadas diferenças nas taxas de infecção em relação à idade, sexo e local de origem das aves.

Wild populations of *Columba livia* Gmelin, 1789 (Columbiformes: Columbidae) are typical inhabitants of urban landscapes, where they are associated with the presence of humans due to the availability of suitable nesting sites and easy access to food, such as human food waste, garbage bins, or intentionally provided by people. These traits have allowed pigeons to successfully expand their range and establish wild populations around the world, particularly in urban settings (Nebel et al., 2020). *Columba livia* is native to Europe, North Africa, the Middle East and South Asia and was introduced to Brazil in the 16th century by European immigrants, being one of the most commonly found birds in urban areas of the city of Pelotas, Rio Grande do Sul (RS), Brazil, where its high population in the environment can increase the exposure of humans and other animals to pathogens (Santos et al., 2020).

Haemosporidian are apicomplexan protozoans transmitted by bloodsucking arthropods. They comprise the most diverse group of blood parasites, composed of the genera *Haemoproteus*, *Leucocytozoon* and *Plasmodium* (Valkiūnas & Iezhova, 2022). *Haemoproteus* are vector-borne avian parasites distributed worldwide, with approximately 177 species described based on gametocyte morphology and peculiarities of their influence on host cells (Valkiūnas & Iezhova, 2022). *Haemoproteus* contains two subgenera that exhibit molecular variations and are associated with distinct vectors: *Haemoproteus* and *Parahaemoproteus,* transmitted by dipterans of the family Hippoboscidae and Ceratopogonidae, respectively (Cepeda et al., 2019; Nebel et al., 2020). These pathogens are considered part of a neglected group of hemoprotozoa, mainly because they rarely damage or cause mortality in birds (Valkiūnas & Iezhova, 2022). However, molecular diagnostics have shown that large-sized megalomeronts develop in many *Haemoproteus* infections, causing damage to various organs. This damage can occasionally lead to severe organ dysfunction or necrosis, with symptoms such as hepatomegaly, hepatitis, hepatic hemorrhage, and necrosis (Lee et al., 2018). These findings indicate that further research is needed to better understand the biology and pathogenesis of haemoproteosis in animals.

*Haemoproteus columbae* Kruse, 1890 (Haemosporida: Haemoproteidae) has been described in *C. livia* and is transmitted by the pigeon louse fly *Pseudolynchia canariensis* (Macquart, 1840) (Diptera: Hippoboscidae) (Cepeda et al., 2019). Studies on hemoproteids in *C. livia* have been conducted worldwide (Waldenström et al., 2002; Tietz Marques et al., 2007; Chagas et al., 2016; Nebel et al., 2020; Rosyadi et al., 2021). In Brazil, investigations in *C. livia* were carried out in the Southeast (Chagas et al., 2016) and South (Tietz Marques et al., 2007) regions, use blood smears and/or molecular techniques as diagnostic methods. Furthermore, the protozoan was detected in wild bird species in the western region of São Paulo, also in the Southeast, through blood smear analysis (Adriano & Cordeiro, 2001). While the blood stages of *Haemoproteus* species are visible in blood smears, species determination is however difficult due to limited number of distinct morphological features (Nebel et al., 2020). Therefore, deoxyribonucleic acid (DNA) sequencing using Polymerase Chain Reaction (PCR) has been established as a more accurate method for identifying lineages and determining parasite prevalence in the blood, especially at low infection intensities (González et al., 2015; Oliveira et al., 2020; Valkiūnas & Iezhova, 2022). Here, we describe for the first time the molecular detection of *H. columbae* in *C. livia* in southern Brazil and determine the prevalence of the haemosporidian by analyzing the infection in relation to the age, sex and place of origin of the birds.

A cross-sectional study was carried out in urban areas of the municipalities of Pelotas (31°46'13”S; 52°20'27”W) and Rio Grande (32°02'45”S; 52°06'15”W), RS, Brazil. This region occupies the extreme south of Brazil and differs significantly from other parts of the country. The fields, characteristic of the Pampa biome, are the predominant landscape and the climate is predominantly humid subtropical, characterized by well-defined seasons and significant temperature variation, with hot summers and harsh winters. Average temperatures vary between 15 and 18 °C, with minimums of -10 °C and maximums of 40 °C. Rainfall is well-distributed throughout the year, with an average precipitation variation of 1,299 to 1,500 mm annually (Rio Grande do Sul, 2024).

A total of 57 free-living pigeons (*C. livia*) (nine immatures and 48 adults) were captured in various locations (e.g., buildings, squares, etc.) in the urban areas of the municipalities studied (21 in Pelotas and 36 in Rio Grande), between May and November 2022. Adult specimens were captured with mist nets, while young pigeons were manually collected from nests. Animals captured were individually placed in cages lined with cotton fabric and transported to the Laboratório de Parasitologia at the Universidade Federal de Pelotas (UFPel) where they were euthanized and necropsied to enable their use in other experiments. During necropsy, the sex of each bird was determined through the observation of their reproductive organs. Euthanasia was performed following the recommendations of Normative Resolution number 1/2013 by the National Council for Animal Experimentation Control (CONCEA). Bird capture, transportation and euthanasia were licensed by the Instituto Chico Mendes de Conservação da Biodiversidade (ICMBio no. 61235-6) and approved by the Ethics Committee for Animal Experimentation (CEEA/UFPel no. 12860/2018).

Blood samples of 57 individual *C. livia* specimens were collected following the procedure described by Cepeda et al. (2019). Blood smears were promptly prepared after collection, and the remaining blood was stored in EDTA buffer at 4 °C until further analysis. Blood smears were fixed with methanol and stained with Giemsa stain following the standard protocol of Samour (2005). An Olympus CX21 light microscope was used to examine the blood smear slides. Initially, a random half of each smear was examined for large extraerythrocytic hematozoan (i.e., *Trypanosoma* and microfilariae) at 200x magnification. Subsequently, the slides were scanned under the microscope at 400x magnification to determine the presence or absence of haemosporidian blood parasites. Approximately 100 fields were screened at low magnification (400x) and 100 fields at high magnification (1000x). Morphological identification was conducted according to Valkiūnas (2004) and Valkiūnas & Iezhova (2022). Digital images of parasites were taken using a digital camera coupled to the microscope at x1000 magnification with an oil immersion objective.

Blood DNA extractions were performed using the commercial PetNAD™ Nucleic Acid Co-Prep Kit (GeneReach Biotechnology Corporation, Taichung City, Taiwan), following the manufacturer's instructions. The quality and quantity of extracted DNA were measured using an ultraviolet light spectrophotometer (Thermo Scientific NanoDrop Lite Spectrophotometer, Waltham, Massachusetts, USA) and 1% agarose gel electrophoresis. The extracted DNA was stored at −20 °C until PCR was performed.

PCR amplification was performed using the primers: HaemF (5'-ATGGTGCTTTCGATATATGCATG-3') and HaemR2 (5'-GCATTATCTGGATGTGATAATGGT-3') which targets an approximately 478 bp fragment of the mitochondrial cytochrome b gene (*CytB*), according to Bensch et al. (2000), with modifications in the thermal profile of the reactions, and was validated using the Basic Local Alignment Search Tool (BLAST). These primers are specific for *Haemoproteus* and *Plasmodium*. In the reactions, 2.0 μL of DNA (50 ng/μL) was used along with a mix containing 2.0 μL of dNTPs (2.5mM), 0.5 μL of each primer (10mM), 2.5μL of buffer solution (10X), 1.25 μL of MgCl2 (50 mM), 0.25 μL of Taq DNA polymerase (5U/μL), and 16.5 μL of ultrapure water, totaling 25 μL. The amplifications, performed in a conventional thermal cycler, included an initial denaturation at 94 °C for 2 minutes, followed by 35 cycles of 94 °C for 45 seconds, 55 °C for 60 seconds, 72 °C for 60 seconds and final extension at 72 °C for 10 minutes. *Plasmodium* spp. DNA kindly provided by the Veterinary Parasitology Laboratory of the Federal University of Santa Maria was used as a positive control, while ultrapure water was used as a negative control. Amplified products were analyzed through electrophoresis using a 1.5% agarose gel, stained with ethidium bromide (0.5μg/mL) and visualized under ultraviolet light. A molecular weight standard of 100pb was used (Ladder 100pb 500µL, Ludwig Biotecnologia, Porto Alegre, Rio Grande do Sul, Brazil). For differentiation of *Haemoproteus* spp. and *Plasmodium* spp., an enzymatic digestion was performed in accordance with methodology previously described by Kistler et al. (2013), and only amplicons compatible with *Haemoproteus* spp. were observed.

Amplicons from two selected samples were excised and purified using a Gel Purification Kit (Ludwig Biotechnology, Porto Alegre, Rio Grande do Sul, Brazil), according to the manufacturer's recommendations, and then sent to sequencing using the BigDye Terminator Cycle Sequencing Kit v3.1 (Thermo Fisher, USA) on an ABI3500 genetic analyzer (Applied Biosystems, USA). Consensus sequences were obtained by electropherogram analysis with Phred base calling and Phrap-assembly tool, and subsequently aligned using MEGA11: Molecular Evolutionary Genetics Analysis version 11 software (Tamura et al., 2021). Multiple sequences were aligned using the ClustalW method and sequence similarity searches were conducted using BLAST with sequences deposited in the National Center for Biotechnology Information (NCBI) and MalAvi databases. Evolutionary history for member species of the Haemoproteidae family was inferred using the Maximum Likelihood method and the General Time Reversible model (Nei & Kumar, 2000). The MEGA11 software was also used to carry out the evolutionary analyses (Tamura et al., 2021). Statistical analysis was performed using the bootstrap method with 1000 repetitions. *Plasmodium falciparum* was used as the outgroup taxon.

Descriptive analyses were conducted using EpiTools epidemiological calculators (Sergeant, 2018). A univariate analysis was performed using Fischer’s exact test to identify the individual characteristics (age and gender) and place of origin of birds associated with parasite presence using the Quantitative Parasitology software (PQweb) (Reiczigel et al., 2019), p-values < 0.05 were considered statistically significant.

Microscopic examination of thin blood smears revealed the presence of haemosporidian parasites in 92.98% (53/57) of *C. livia* specimens in this study. Intraerythrocytic stages of hemosporids were observed, with mature gametocytes exhibiting a halteridial position, appearing sausage-shaped, and in contact with the nuclei and envelope of erythrocytes.

**Figure 1:**
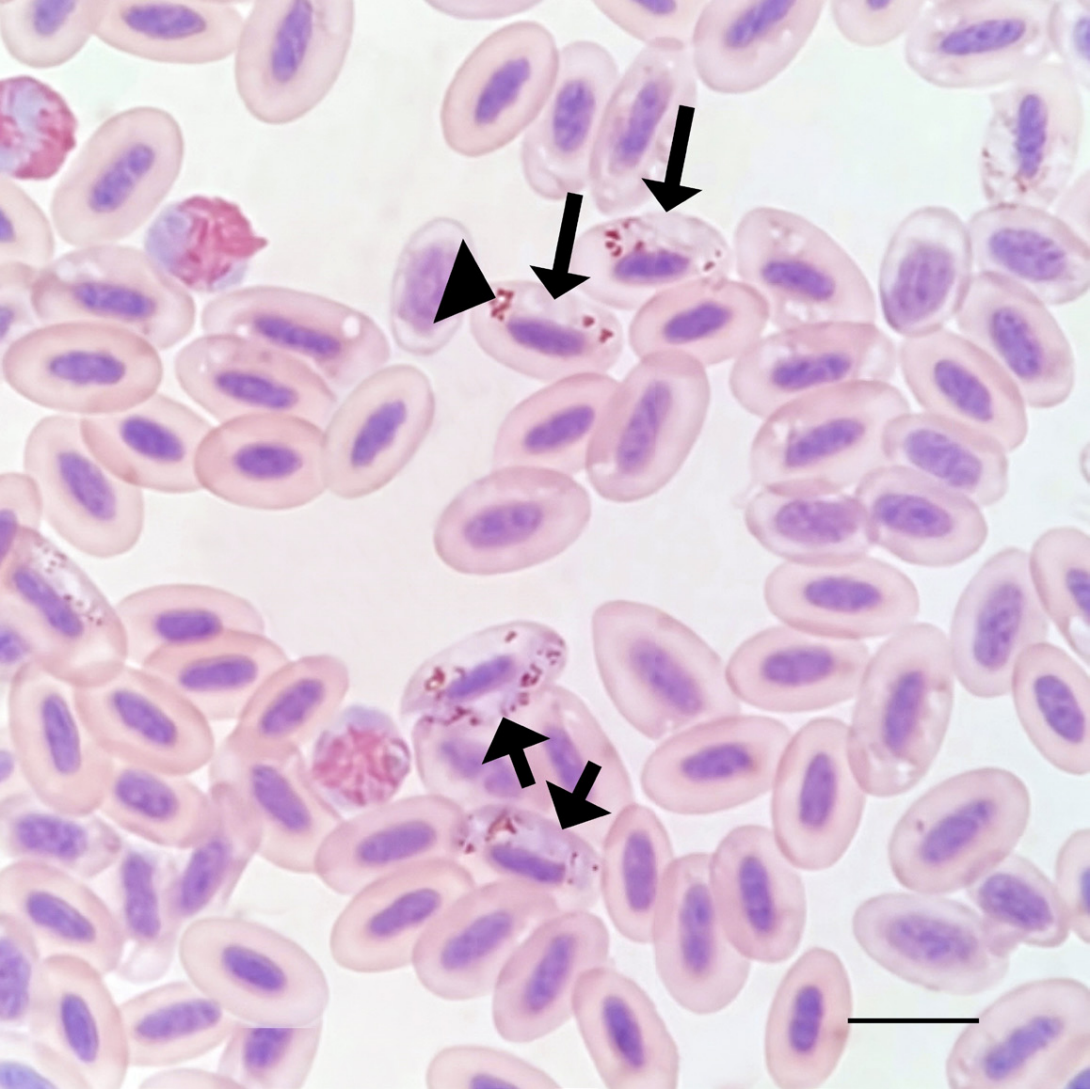
*Haemoproteus columbae* from the blood of *Columba livia* in southern Brazil. Macrogametocytes (short simple arrows) and microgametocytes (long simple arrows) are shown. Note the aggregation of pigment into large compact masses in microgametocytes (arrowheads). Giemsa stained thin blood films. Bar = 10μm.

Additionally, the haemosporidians pigment granules (hemozoin) were clearly visible, especially in microgametocytes, with a notable aggregation into large compact masses (Figure 1) a distinctive feature of *H. columbae* (Valkiūnas, 2004; Valkiūnas & Iezhova, 2022). The overall results of positivity for *H. columbae* are shown in Table 1. There were no significant statistical differences found across the analyzed variables.

**Table 1:** Prevalence (%) of *Haemoproteus columbae* observed on *Columba livia* in relation to age, gender and place of origin. Blood samples were collected between May to November 2022, in the municipality of Pelotas and Rio Grande, Rio Grande do Sul, Brazil.

**Independent variables**	**N**	**Frequency**	**CI**	**p-value**
**Age**				
Juvenile	9	88.89%	56.5-98.01%	0.5215
Adult	48	93.75%	83.16-97.85%	
**Gender**				
Female	18	94.44%	74.24-99.91%	0.6037
Male	37	91.89%	78.7-97.2%	
Undefined gender	2	-	-	
**Place of origin**				
Pelotas	19	90.48%	71.09-97.35%	0.4713
Rio Grande	34	94.44%	81.36-98.46%	

N = number of samples; CI = confidence interval.

**Figure 2:**
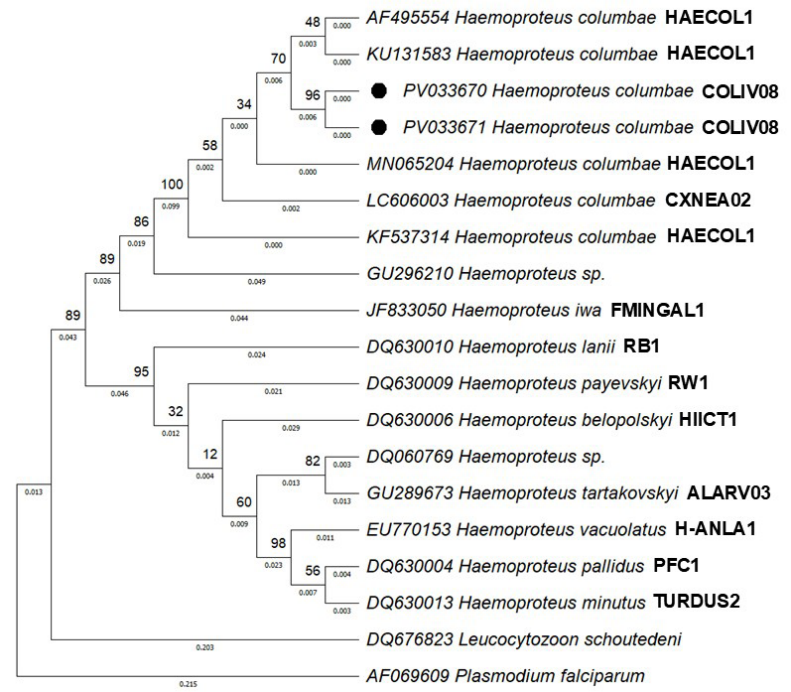
Phylogenetic tree for members of the Haemoproteidae based on *CytB* gene sequences and inferred using the Maximum Likelihood method and the General Time Reversible model. GenBank accession numbers for all sequences are given in front of the taxon names, while known lineages are given behind the taxon names. The black circles represent the samples from this study. The bootstrap consensus tree was inferred from 1000 replicates. *Plasmodium falciparum* was used as an outgroup taxon. Probability values ​​are represented by the numbers at the nodes.

To confirm the identification of the parasite, positive samples were subjected to molecular analysis. All samples that were positive by blood smear were also confirmed positive in the PCR analysis, indicating a high prevalence of haemosporidian parasites in these birds. The sequences obtained for the *CytB* gene were deposited in NCBI GenBank under accession numbers PV033670 and PV033671 (Figure 2). Using the BLAST tool, all sequenced samples were compared with previously deposited sequences in the GenBank, confirming the presence of the target protozoan’s DNA. The sequences obtained showed 98.87-99.08% similarity with *H. columbae* (GenBank accessions AF495554, KU131583, MN065204, LC606003 and KF537314). All obtained sequences clustered with the subgenus *Haemoproteus (Haemoproteus).* No parasites of the genus *Plasmodium* or any other hematozoans were detected.

In the molecular analysis, we observed that our sequences correspond to a new strain of *H. columbae*, deposited in the MalAvi database and identified as COLIV08. This lineage showed 99% similarity to lineages HAECOL1, COQUI05, COLPAL02, COLIV07, CXNEA02, COLIV03 and AFR112.

The phylogenetic analysis inferred using *CytB* gene sequences (Figure 2) showed that the two *H. columbae* isolates in this study formed a clade with *H. columbae* isolated from *C. livia* from a zoo in São Paulo, Brazil (lineage HAECOL1) (GenBank accession KU131583) (Chagas et al., 2016), as well as isolates from Colombia (lineage HAECOL1) (GenBank acc. n. KF537314) (González et al., 2015), Botswana, Southern Africa (lineage HAECOL1) (GenBank acc. n. AF495554) (Waldenström et al., 2002), Central Java, Indonesia (lineage CXNEA02) (GenBank acc. n. LC606003) (Rosyadi et al., 2021) and Cape Town, South Africa (lineage HAECOL1) (GenBank acc. n. MN065204) (Nebel et al., 2020). *Haemoproteus* sp. (GenBank acc. n. GU296210) and *Haemoproteus iwa* (lineage FMINGAL1) (GenBank acc. n. JF833050) were recovered in sister clades.

*Haemoproteus* and *Plasmodium* species do not digest hemoglobin completely, resulting in the accumulation of residual pigment (hemozoin) in their blood stages, this feature distinguishes them from species of the genus *Leucocytozoon*, which do not produce residual pigment (Valkiūnas & Iezhova, 2022). However, due to morphological similarity among species, differentiation based solely on blood smears can be challenging. Therefore, both morphological and molecular data complement each other and are essential to achieve a better understanding of parasite diversity.

In this study, we present morphological and molecular evidence of hemoparasites of the species *H. columbae* in free-living pigeons (*C. livia*) from southern Brazil. The blood smear screenings showed a haemosporidian infection rate of 92.98%, indicating a high prevalence of these parasites in the studied population. This is comparable to previous Brazilian studies that reported total prevalence of *H. columbae* of in *C. livia* (Chagas et al., 2016) and in *Zenaida auriculata* Des Murs, 1847 (Adriano & Cordeiro, 2001) from São Paulo. High prevalences are expected in these columbids, as they live in large flocks, facilitating the transmission of vectors, and consequently the protozoan (Nebel et al., 2020). However, lower prevalence have been observed in the columbids *Columbina talpacoti* Temminck, 1811 in the northeast (2.29%) (Lugarini et al., 2018) and in the southeast region of the country (51.6% in *C. talpacoti* and 19.3% in *Columbina squammata* Richmond, 1896) (Adriano & Cordeiro, 2001). The high prevalence of the protozoan in this study is noteworthy, as *C. livia* is an invasive and widely distributed species that could facilitate the spread of its parasites to new hosts and potentially threaten native species (Chagas et al., 2016; Nebel et al., 2020), since the host specificity and geographic distribution of Haemosporidian species may vary (Oliveira et al., 2020).

The prevalence and distribution of infection by Haemosporidian species vary by region, bird order, and transmission vector. Previous observations have shown that *Haemoproteus* species vary in their specificity to vertebrate hosts but typically fail to complete their life cycle and form gametocytes in birds of different orders, resulting in abortive (incomplete) development (Valkiūnas & Iezhova, 2022). *Columba livia* is commonly associated with *H. columbae*, a relationship that can be explained by the ecology and biting behavior of pigeon louse fly (*P. canariensis*), with adult stages virtually flightless, crawling on the host body surface, being substantially different from mosquitoes, which have a wider host range (Rosyadi et al., 2021). In this study, *P. canariensis* fly was found beneath the feathers of most birds. In a related study from the same region, *P. canariensis* was observed in both juveniles and adults of *C. livia*, with higher levels of infestation noted in warm seasons (Amaral et al., 2017).

Other factors such as age and gender has been associated with *H. columba*e infection. In this study, the prevalence of *H. columbae* infection in female and male pigeons was similar (p = 0.6037), consistent with the findings from Asia by Adinehbeigi et al. (2018). Resistance to haematozoan infections could be related to gender-specific interactions with environmental conditions, furthermore, differences in infection intensity and incidence between males and females are related to stress and immunosuppression and are associated with the physiological state of the hosts as well as the ecology of the vectors (Adinehbeigi et al., 2018). However, natural behaviors can contribute to equal exposure to vectors. Males and females of *C. livia* show similar behaviors, moving and feeding together and performing equal parental functions (Ramos-Gorbeña et al., 2021). Regarding age, no statistical difference was observed in infection rates between immature and adult birds, contrary to findings from a study in Iran (Adinehbeigi et al., 2018), where younger birds (1 to 6 months old) showed higher infection rates and were 1.539 times more susceptible to infection by *H. columbae*. Higher prevalences in younger birds may be related to vector exposure and/or low immunity (Adinehbeigi et al., 2018), but this was not evaluated in our study. Although no associations between age and sex and infection were observed, it should be considered that the sample size may not be sufficient to identify more subtle relationships. Further studies are needed to better understand host-parasite relationships.

Finally, this is the first molecular detection of *H. columbae* in *C. livia* in Southern Brazil and haemosporidian were found at a high prevalence (92.98%) in the study population. No differences in infection rates were observed regarding age, gender or place of origin of the birds. A new lineage of the protozoan was found, indicating that the diversity of lineages may be even greater in the region, since a low number of samples were sequenced in this study. Research on introduced birds is critical due to their potential to spread parasites to native birds, therefore, additional studies are needed to understand the real genetic diversity of the parasite in the study region.
